# The long non-coding RNA FLJ46906 binds to the transcription factors NF-κB and AP-1 and regulates expression of aging-associated genes

**DOI:** 10.18632/aging.101528

**Published:** 2018-08-17

**Authors:** Kazuyuki Yo, Thomas M. Rünger

**Affiliations:** 1 Department of Dermatology, Boston University School of Medicine, Boston, MA 02118, USA; 2 Current address: Dermatological R & D, POLA Chemical Industries Inc., Yokohama, Japan

**Keywords:** long non-coding RNA, aging, skin, NF-κB, AP-1, FLJ46906

## Abstract

Several features differentiate aged cells from young cells, many of which are due to changes in gene expression during the aging process. The mechanisms of altered gene expression in aging cells remain incompletely understood, and we hypothesized that long non-coding (lnc) RNAs mediate at least some of these changes. We screened for alterations in lncRNA expression with aging in skin fibroblasts and identified the lncRNA FLJ46906 to be consistently upregulated with aging in-vivo and in-vitro. The function of this lncRNA has not been known. Here we show that FLJ46906 regulates several aging-associated genes, including *IL1B*, *IL6*, *CXCL8*, *TGFB1,* and *ELN.* We suggest that these effects are mediated through NF-κB and AP-1, because these aging-associated genes are regulated by NF-κB and AP-1, and because we found that FLJ46906 directly binds to these two transcription factors. This data supports a role of the lncRNA FLJ46906 in the aging process.

## Introduction

More than 93% of the human genome is transcribed [1]. Most of the transcripts are non-coding RNAs, including microRNAs, piwi-interacting RNAs, small nucleolar RNAs, and long non-coding RNAs (lncRNA) [2]. LncRNAs are more than 200 nucleotides in length, expressed in a tissue- and cell type-specific manner, and are classified into six groups based on their genomic locus in relation to neighboring genes (antisense, intergenic, overlapping, intronic, bidirectional, and processed) [3]. One of the best characterized lncRNAs is *XIST*, known to mediate silencing of the X-chromosome by inducing recruitment of the polycomb repressive complex [4]. Other lncRNAs have been reported to regulate gene expression at the transcriptional or post-transcriptional level [5,6]. The GENCODE v7 catalogue of human long non-coding RNAs lists a total of 14880 lncRNAs, but there are likely more than that [7]. More recently, the total number of lncRNAs has been suggested to be close to 60,000 [8]. Of those, the Reference Database for Functional Long Noncoding RNAs, maintained by the Genome Informatics Group, lists only 181 human lncRNAs for which a function has been described [9], indicating that the function of the vast majority of lncRNAs is unknown.

Many different features characterize aging of an organism, including tissue dysfunction, inflammation, and occurrence of age-associated diseases. On the cellular and molecular level, aging is characterized by loss of synthetic functions and proliferative capacity, senescence, accumulation of abnormal dysfunctional macromolecules, accumulation of DNA damage and mutations, telomere shortening, and changes in cellular morphology with increases in cell size. All of these alterations are accompanied by changes in the expression of genes. Many of the aging-associated changes in gene expression are cell- or tissue-specific, and only a few are observed across different types of cells and tissues [10,11]. With aged dermal fibroblasts, changes have been reported for genes encoding cytokines, extracellular matrix proteins, and matrix metalloproteinases, sometimes called aging-associated genes [12–15].

The mechanisms by which expression of genes changes with aging are poorly understood. Given the increasingly recognized role of lncRNAs in the regulation of gene expression, we hypothesized that lncRNA play important roles in mediating aging-associated changes in gene expression.

## RESULTS

### Expression of the lncRNA FLJ46906 increases with aging in fibroblasts

**Figure 1:**
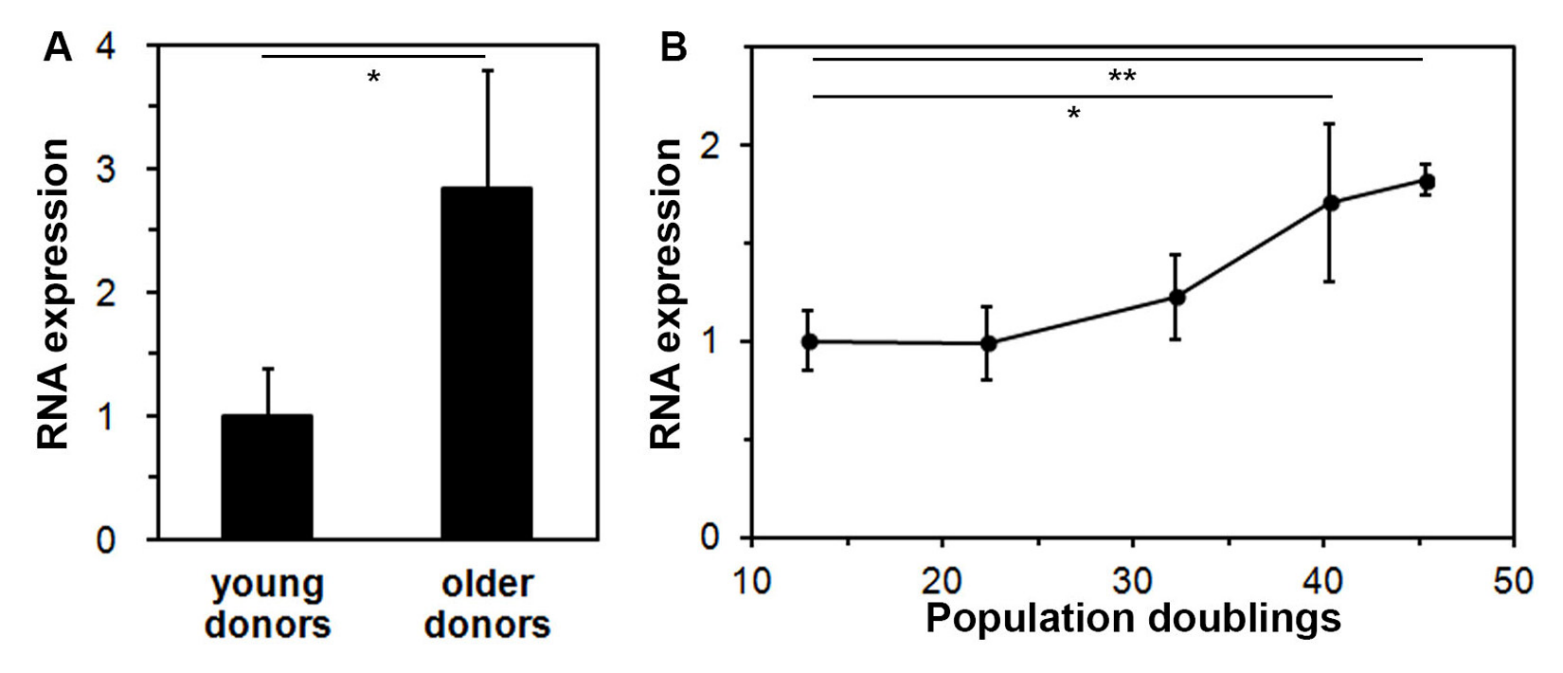
**Expression of the lncRNA FLJ46906 increases with aging in fibroblasts.** (**A**) The expression of FLJ46906 in fibroblasts from older donors (63, 68, and 70 years old, aged *in-vivo*), as measured by qPCR, is 2.8 fold higher than in fibroblasts from young donors (18, 23, and 27 years old; n = 3 (triplicate samples from each donor), mean ± SD, *p < 0.05). (**B**) Neonatal fibroblasts aged *in-vitro* by longterm culture show increasing expression of FLJ46906 with increasing population doublings (PD), as measured by qPCR (n = 3 (cells from triplicate tissue culture dishes), mean ± SD, *p < 0.05, **p < 0.01).

To discover lncRNAs that are involved in the aging process, expression of lncRNAs was compared between fibroblasts from a young, 23 year-old donor and an older, 70 year-old donor using DNA microarrays (SurePrint GE3 Human Gene Expression 8X60K Microarray Kit). Out of approximately 7,000 probes for lncRNAs on the microarray, a more than 2-fold higher expression in the older fibroblasts was found for 28 lncRNAs, and a more than 2-fold lower expression in 35 lncRNAs. The entire microarray data set can be accessed at https://www.ncbi.nlm.nih.gov/geo/query/acc.cgi?acc=GSE117545. The sequence of twelve of these lncRNAs could be found and validated in the NCBI Human Genome Reference Sequence Database [16]. To confirm these microarray data, expression of these twelve lncRNAs was studied in cells from three young (18, 23, and 27 years old) and three older donors (63, 68, and 70 years old) using quantitative PCR. Only one lncRNA, FLJ46906, showed a consistent and significantly higher expression in the fibroblasts from the three older donors, as compared to the expression levels in the cells of the three young donors (Figure 1A).

To further investigate upregulation of FLJ46906 with aging, we used an in-vitro aging model, for which we continuously cultured neonatal fibroblasts with documentation of cell counts at each passing for determination of population doublings. Fibroblasts harvested at various time points showed increasing expression of FLJ46906 with increasing population doublings (Figure 1B).

### The lncRNA FLJ46906 does not regulate the expression of neighboring genes

**Figure 2:**
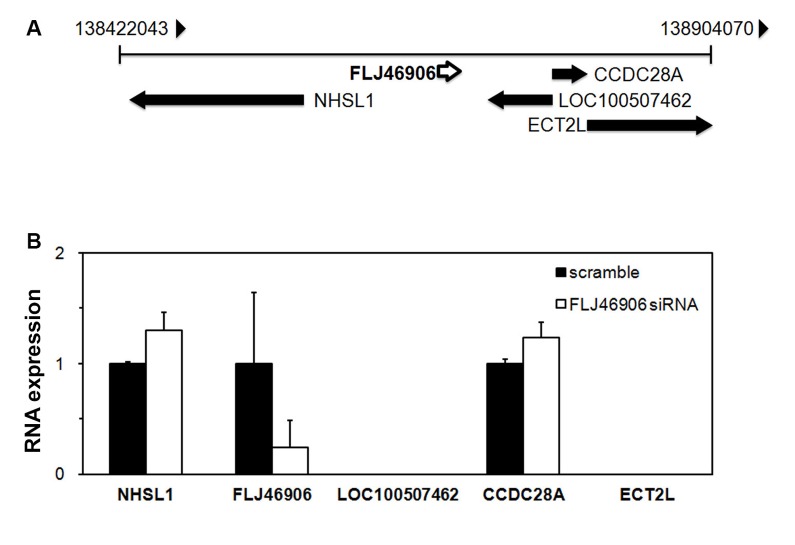
**The lncRNA** **FLJ46906 does not regulate the expression of neighboring genes.** (**A**) Genome map around FLJ46906 gene locus on chromosome 6. (**B**) The expression of two of the FLJ46906’s neighboring genes is not affected by knockdown of FLJ46906, as determined by qPCR; two other neighboring genes are not expressed in neonatal fibroblasts (n = 3, mean ± SD).

As for most lncRNAs, the function of FLJ46906 has not been described yet. Some lncRNAs regulate expression of neighboring genes (in cis), others of more distant genes (in trans) [17]. To address whether FLJ46906 regulates the expression of its neighboring genes in cis, the expression of the four neighboring genes, as identified through the NCBI Reference Sequence Database, NHSL1, CCDC28A, LOC100507462, and ECT2L (Figure 2A) was determined after knockdown of FLJ46906 expression. Expression of NHSL1 and CCDC28A was not affected by the effective knockdown of FLJ46906, indicating that they are not regulated by FLJ46906 (Figure 2B). Using two different pairs of PCR primers, expression of LOC100507462 and ECT2L was not detectable in the fibroblasts, excluding these genes as potential targets of FLJ46906 in these cells as well.

Some lncRNAs can regulate gene expression by binding to the mRNA of other genes (anti-sense mechanism), either in the vicinity of its gene locus, or anywhere else in the human genome, similar to siRNA. We therefore searched the human genome database for homologies with the antisense sequence of FLJ46906. No such homologies were found anywhere in the human genome, indicating that the effect of FLJ46906 on the expression of other genes is not mediated via an anti-sense mechanism.

### Aging-associated genes are regulated by the lncRNA FLJ46906

Since FLJ46906 expression increases with aging in fibroblasts, we hypothesized that it regulates aging-associated genes. After knockdown of FLJ46906, we therefore assessed the expressions of several coding genes that are well known to change with aging and represent three different processes that are well known to be altered with aging; inflammation/senescence-associated secretory phenotype (*IL1B, IL6, CXCL8, TGFB1*), metabolism of the extracellular matrix (*ELN, COL1A1, MMP1, MMP3, MMP9, MMP14*), and cell cycle regulation (*CDKN1A*). Several lncRNAs have been associated with aging-associated conditions such as diabetes, cancer and mitochondrial dysfunction. However, here we focused on genes and processes associated with skin aging, as our model system is the skin-derived fibroblast.

**Figure 3:**
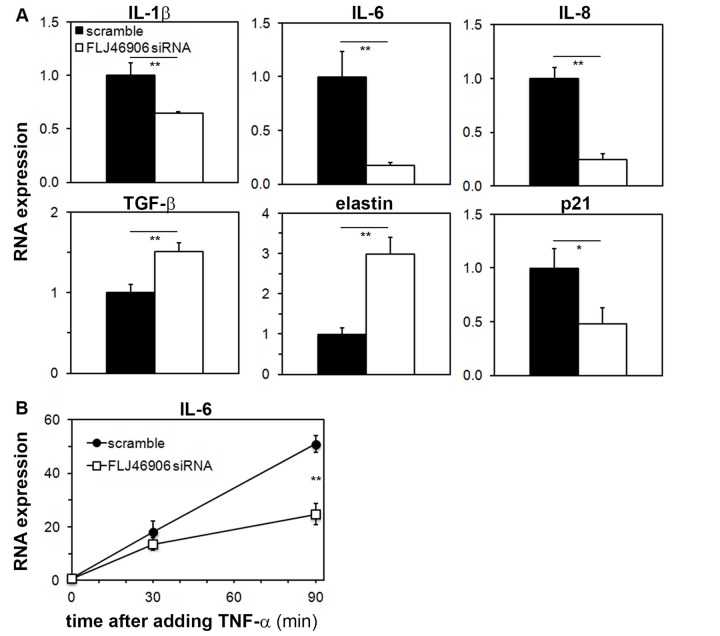
**Aging-associated genes are regulated by the lncRNA FLJ46906*.*** (**A**) Baseline expression of *IL1B* (interleukin-1β), *IL6* (interleukin-6), *CXCL8* (interleukin-8), *TGFB* (transforming growth factor-b), and *ELN* (elastin), as determined by qPCR, is altered by knockdown of FLJ46906 (n = 3, mean ± SD, *p < 0.05, **p < 0.01). (**B**) The induction of IL-6 by TNF-α (20 ng/ml; R&D Systems) is partially inhibited by knockdown of FLJ46906 (n = 3, mean ± SD, **p < 0.01).

The baseline expression of inflammatory cytokines *IL1B* (interleukin-1β) and *IL6* (interleukin-6), and of the chemokine *CXCL8* (interleukin-8) were significantly downregulated, while *TGFB1* (transforming growth factor*-*β) and *ELN* (elastin) were upregulated with knockdown of FLJ46906 (Figure 3A). Repeat experiments with a completely different siRNA to knock down FLJ46906 showed similar results (Supplementary Figure 1). Expression of *COL1A1* (Collagen type I, alpha 1), matrix metalloproteinases *MMP1*, *MMP3*, *MMP9* and *MMP14, and CDKN1A* (p21) was not altered (data not shown).

In order to investigate whether FLJ46906 also affects induced gene expressions, as opposed to just baseline, steady-state expressions, we induced expression of IL-6 with TNF-α and measured the induction of IL-6 with and without knockdown of FLJ46906. IL-6 was chosen, as its expression is well known to be highly variable and inducible. TNF-α is a major regulator of IL-6 expression. After binding to its receptor, it activates NF-κB, a key mediator of inflammatory and stress responses, well known to transcriptionally induce IL-6. With that, the induction of IL-6 by TNF-α is a good system to investigate the behavior of NF-κB. The up to 50-fold induction of IL-6 by TNF-α was significantly abrogated by knockdown of FLJ46906, indicating that reduced levels of FLJ46906 not only affect baseline expression of IL-6, but also reduce induced expression of IL-6 (Figure 3B).

### The lncRNA FLJ46906 binds to the transcription factors NF-κB and AP-1

All aging-associated genes whose expression was changed with knockdown of FLJ46906 are known to be regulated by the inflammatory transcription factors NF-κB and AP-1 [18–28], suggesting that FLJ46906 may regulate the expression of aging-associated genes by affecting function of these transcription factors. Possible mechanisms could include:

1) regulation of the two transcription factors’ expression by FLJ46906,

2) interference of FLJ46906 with activation of the transcription factors or their trafficking to the nucleus,

3) binding of FLJ46906 to the two transcription factors’ DNA binding sites,

4) interference of FLJ46906 with the recruitment of the transcription factors to their binding sites,

5) or binding of FLJ46906 to NF-κB and AP-1 directly.

To address the first possible mechanism, expression of NF-κB and AP-1 was measured with and without knockdown of FLJ46906. Neither the mRNA, nor the protein levels of NF-κB and AP-1 were changed by knockdown of FLJ46906, indicating that FLJ46906 does not regulate the expression of these transcription factors (Supplementary Figure 2).

Various stimuli, including e.g. longwave ultraviolet light (UVA) or TNF-α activate NF-κB and AP-1 [29,30]. An important step in the activation of NF-κB is the release from its binding to the cytoplasmic IκB, which then enables translocation to the nucleus. Immunoprecipitation between NF-κB and IκB was not altered following knockdown of FLJ46906, indicating that FLJ46906 does not interfere in this step of the activation of NF-κB (Supplementary Figure 3A). AP-1 is activated by phosphorylation. Knockdown of FLJ46906 did not alter this phosphorylation (Supplementary Figure 3B), ruling out a role of FLJ46906 in this step of AP-1 activation.

In order to investigate whether FLJ46906 affects translocation of NF-κB and/or AP-1, we used cells with and without knockdown of FLJ46906 and compared trafficking of NF-κB and AP-1 following irradiation with UVA. For NF-κB, translocation from the cytosol into the nucleus was observed within 45 minutes after UVA exposure. After three hours, NF-κB was again observed in the cytosol, indicating translocation from the nucleus back to the cytosol. This trafficking pattern was not changed in FLJ46906 knockdown fibroblasts (Supplementary Figure 4, upper panels). AP-1 was located in both nucleus and cytosol in non-stimulated fibroblasts. 1.5 hours after UVA exposure, cytosolic AP-1 was translocated into the nucleus. After six hours, it was again observed in the cytosol. The same pattern was observed in FLJ46906 knockdown fibroblasts (Supplementary Figure 4, lower panels). This data suggests that FLJ46906 does not affect the trafficking of NF-κB and AP-1 between the nucleus and the cytosol.

In order to investigate possible binding of FLJ46906 to the DNA binding sites of NF-κB and/or AP-1, we searched the sequence of FLJ46906 for sequences that would be anti-sense to the NF-κB binding site sequences (“GGGRNYYYCC”) or the AP-1 binding site sequences (“TGA(C/A)T(C/A)A”, including TRE: TGACTCA, A-TRE: TGAATCA, and AA-TRE: TGACTAA). No such anti-sense sequences were found on FLJ46906, indicating that FLJ46906 cannot directly bind to the DNA binding sites of NF-κB or AP-1.

Some lncRNAs are known to regulate access of transcription factors to their binding sites by affecting chromatin remodeling [17] and we hypothesized that FLJ46906 has similar effects and alters recruitment of NF-κB and AP-1 to their binding sites. After incubation with TNF-α to induce recruitment of NF-κB to its DNA binding site, we used the chromatin immunoprecipitation (ChIP) assay to compare binding of NF-κB to the IL-6 promoter in cells with or without knockdown of FLJ46906. The scramble oligo-transfected fibroblasts showed drastic increase of NF-κB on its binding site after addition of TNF-α, but this enrichment of NF-κB was not changed in FLJ46906 knockdown fibroblasts (Supplementary Figure 5). This indicates that FLJ46906 does not affect the recruitment of NF-κB to its binding site on the IL-6 promotor. We also used the ChIP assay to measure binding of AP-1 to its binding site on the IL-6 promoter. Unlike other reports that demonstrate such binding following stimulation with TNF-α, e.g. in ovarian-carcinoma cells [31], we did not observe it in fibroblasts, either at baseline or after stimulation (data not shown).

**Figure 4:**
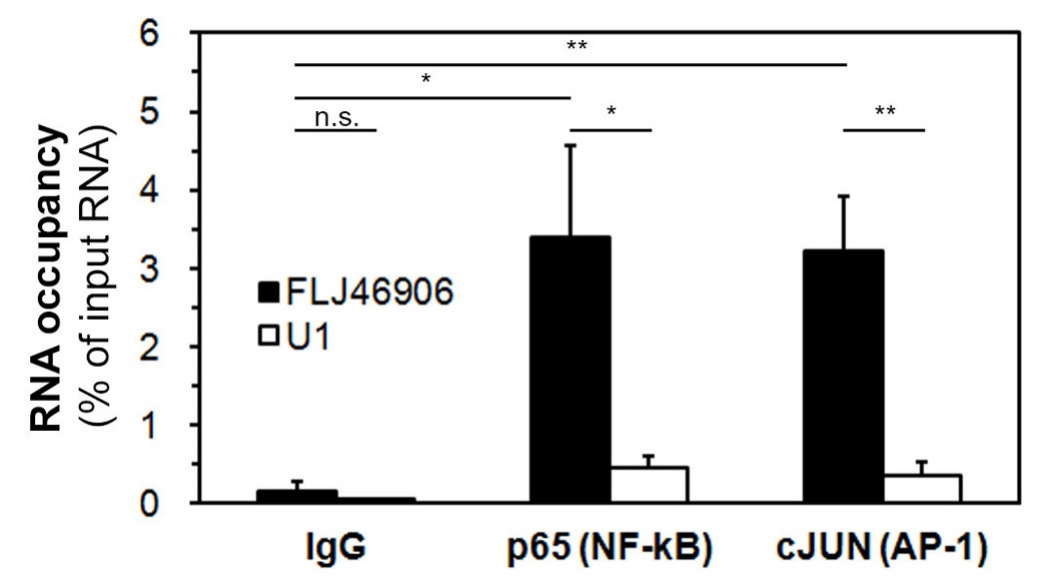
**The lncRNA FLJ46906 binds to the transcription factors NF-κB and AP-1.** FLJ46906 was amplified using qPCR after immunoprecipations with antibodies against p65 (NF-κB) or cJUN (AP-1). U1 = negative RNA control; IgG = negative protein control (n = 3 independent experiments, triplicate samples each; mean ± SEM, *p < 0.05, **p < 0.01).

It has been reported that some lncRNAs regulate transcription by directly binding to transcription factors or transcription-regulating proteins, such as SMAD2/3, p300 or PRC2 [17,32,33]. In order to address whether FLJ46906 can bind to NF-κB and AP-1 directly, RNA immunoprecipitation assays were performed. After immunoprecipitating RNA-protein complexes using antibodies directed against NF-κB or AP-1, the amount of FLJ46906 RNA was quantified by qPCR (Figure 4). U1 (negative RNA control, not expected to bind to NF-κB or AP-1) showed only marginal enrichment. Binding of FLJ46906 to NF-κB and AP-1 was significantly higher than the binding of U1 RNA, but also significantly higher than the IgG-immunoprecipitated sample (negative protein control). This result indicates that FLJ46906 binds to both NF-κB and AP-1.

## DISCUSSION

Using both in-vitro and in-vivo aged cells, we here show that the lncRNA FLJ46906 is upregulated in aged skin fibroblasts. Other groups have also investigated changes in lncRNA expression during aging. Using long-term cultures (an in-vitro aging model) of WI-38 lung fibroblasts and comparing early passage cells with senescent cells, Abdelmohsen et al. [34] observed changes in the expression of a large number of lncRNAs, but not of FLJ46906. In contrast to Abdelmohsen et al., we did not explicitly study senescent cells. Instead, we used aged cells that were mostly still proliferating. This may explain the difference in results, because expression profiles of aged, but still proliferating cells are different from those of fully senescent cells. However, our cells also had an increased expression of FLJ46906 when they stopped growing past 46 population doublings (last data point in Fig 1B), indicating that our cells also have an increased expression of FLJ46906 when senescent. The difference between their and our results may also be explained by the use of different cell types, our primary skin fibroblasts vs. their fetal, fibroblast-like lung cells. Glass et al. [10] studied the expression profiles in whole skin taken from young and older donors and also did not observe a significant change in the expression of FLJ46906. The vast majority of cells in whole skin samples, however, consist of keratinocytes, as the density of fibroblasts is dramatically lower in the dermis than the density of keratinocytes in the epidermis. We therefore suggest that the fact that Abdelmohsen et al. [34] and Glass et al. [10] did not find a change in FLJ46906 expression in senescence or aging is due to differences in lncRNA regulation between tissue and cell types. Such differences have indeed been described for many other lncRNAs [35]. On the other hand, the expression of FLJ46906 is quite ubiquitous, as it has been described in brain, spleen, and 22 other tissues [36]. It is therefore conceivable that the association of FLJ46906 with aging may not be limited to the cell type studied by us, the dermal fibroblasts, but also characterize aging of other cell types.

The lncRNA FLJ46906 was first described by the Mammalian Gene Collection Program Team [37], but its function has remained unknown. Some lncRNAs, e.g. *XIST* and *ANRIL*, regulate genes in their immediate vicinity (cis-acting), but this we found is not the case with FLJ46906. Instead, we found that it up-regulates some distant genes that are known to be upregulated in aging (*IL1B*, *IL6*, *CXCL8*) and that it downregulates some distant genes that are known to be downregulated in aging (*TGFB1,* and *ELN)*. All of these genes are regulated by the transcription factors NF-κB and AP-1. The reported effects of NF-κB activation, increased expression of *IL1B*, *IL6*, *CXCL8* and reduced expression of *TGFB1,* and *ELN* completely match the direction of gene expression changes with FLJ46906. This is also true for AP-1. This suggested a possible role of FLJ46906 in the function of NF-κB and AP-1.

First, we excluded several possibilities how FLJ46906 could alter the activity of NF-κB and/or AP-1, including direct alteration of their expression, activation, trafficking, or recruitment to DNA binding sites. We then found that FLJ46906 directly binds to NF-κB and to AP-1.

There are several prior reports showing that a lncRNA can regulate the activity of a transcription factor: Jiang et al. [32] described the direct binding of the lncRNA DEANR1 to the transcription factor SMAD2/3, and that this binding regulates the activity of SMAD2/3. Lnc-DC, a lncRNA exclusively expressed in dendritic cells, was shown to bind to the transcription factor STAT3 and to promote STAT3 phosphorylation [38]. The lncRNA RMST binds to the transcription factor SOX2 and with that mediates the recruitment of SOX2 to its DNA binding sites in neural cells [39]. Using breast cancer cell lines, Liu B et al. [40] describe a lncRNA they call NKILA for NF-κB interacting lncRNA, which is not only induced by NF-κB, but also interacts with the NF-κB /IκB complex to prevent overactivation of NF-κB. Parts of this report have been questioned with an alternative explanation being put forward that NKILA exerts its effects through its antisense properties to PMEPA1. In addition, there are several more reports on lncRNAs regulating the gene expression of transcription factors [41–43].

We show that FLJ46906 is another transcription factor-binding lncRNA and suggest that the alteration of the aging-associated genes by FLJ46906 is mediated by the binding of FLJ46906 to NF-κB and/or AP-1. How exactly the binding of FLJ46906 to these transcription factors affects their activity remains unclear. There are numerous binding partners to these factors that regulate their activity [44,45], and one of these interactions may be altered by binding of FLJ46906. Alternatively, the binding may directly affect its transcription-initiating activity.

Although many lncRNAs appear to be associated with aging or senescence [34], FLJ46906 is one of very few aging-associated lncRNAs for which a molecular mechanism has been elucidated. One other example is the lncRNA ANRIL, which represses the expression of p16 [46].

Asking the question what causes the changes in gene expression during the aging process, we can now state that the lncRNA FLJ46906 mediates the changes in the expression of some aging-associated genes in skin fibroblasts. This raises the question what causes the change in the expression of FLJ46906 during aging. At this time, this question remains unanswered.

## MATERIALS AND METHODS

### Cell culture, transfection, and UVA irradiation

Adult primary human dermal fibroblasts established from abdominal skin of donors aged 18, 23, 27, 63, 68, and 70 years were purchased from Kurabo (Japan). Neonatal fibroblasts were established from neonatal foreskin using standard protocols [47]. Cells were used with passage numbers less than 10, unless otherwise noted and were cultured in Dulbecco's Modified Eagle's Medium (Thermo Fisher Scientific) supplemented with 10% fetal bovine serum and 1% penicillin/streptomycin at 37°C and 5% CO_2_. SiRNA to inhibit expression of FLJ46906 (Supplementary Table 1) was purchased from Qiagen and transfected using lipofectamine 3000 (Thermo Fisher Scientific) according to the manufacturer’s protocol. For irradiation with longwave ultraviolet light (UVA), cells were washed twice with phosphate-buffered saline (PBS) and then irradiated through a thin cover of PBS using the metal-halide Sellamed 1200 UVA lamp from Sellas Sunlight (emission 328 – 460 nm, emission maximum at 370 nm).

### Gene expression analysis

For RNA expression analysis, total RNA was isolated from fibroblasts using TRIzol (Thermo Fisher Scientific). After reverse transcription using iScript Reverse Transcription Supermix for RT-qPCR (BioRad), cDNA was amplified by iTaq Universal SYBR Green Supermix (Bio-Rad) using the CFX96 Real-Time System (BioRad). Primers are listed in the Supplementary Table 1

For protein expression analysis, western blotting was performed with antibodies against p65 (Abcam), cJUN (Abcam), and β-actin (Lifetechnologies).

### RNA immunoprecipitation assay

To demonstrate binding of the lncRNA to proteins, the RNA immunoprecipitation assay was used as described previously [17] with minor modifications.

Briefly, the nuclear fraction of cells was harvested using a nuclear isolation and lysis buffer (1.28 M sucrose, 40 mM Tris-HCl (pH 7.5), 20 mM MgCl_2_ and 4% Triton X-100) followed by centrifugation. After resuspending in RIP buffer (150 mM KCl, 25 mM Tris (pH 7.4), 5 mM EDTA, 0.5 mM DTT, 0.5% NP40, 100 U/mL SUPERase In RNAase inhibitor (Invitrogen), 1 X cOmplete ULTRA (Roche)), chromatin was sheared by sonication. Dynabeads (Protein G, Lifetechnologies) were loaded with antibodies against p65 or cJUN by incubation at 4°C for three hours and then transferred to chromatin-sheared cell lysates for overnight incubation. After washing with RIP buffer, TRIzol was added to isolate RNA from RNA-protein complexes. Finally, reverse-transcription and qPCR were performed as above to amplify RNA that was bound to p65 or cJUN.

### Detection of protein trafficking

After irradiation of fibroblasts grown on glass coverslips with UVA, immunocytochemistry was performed using standard procedures (fixation with 3% paraformaldehyde; permeabilization with 0.5% Triton X-100, blocking with 1% BSA). The same antibodies as those mentioned above for the Western blotting were used for the primary immunoreaction. An IgG rhodamine-conjugated secondary antibody (Thermo Fisher Scientific) was used for labeling. Nuclei were counterstained with DAPI (Sigma-Aldrich).

### Chromatin immunoprecipitation (ChIP) assay

Fibroblasts were fixed by 0.75% formaldehyde for 10 minutes for cross-linking between proteins and DNA, followed by incubation with 125 mM glycine for 5 minutes to stop fixation. After harvesting cells with ice-cold PBS, chromatin was sheared by sonication. Magnetic protein G beads (Thermo Fisher Scientiic) and antibody against p65 (abcam) or IgG were transferred to the sheared-chromatin and incubated in a rotator at 4°C for 16 hours. After washing three times, reverse cross-linking was performed by heating at 65°C for 4 hours, followed by phenol-chloroform extraction to purify DNA. Enrichment of DNA was determined using qPCR. Primers are listed in Supplementary Table 1.

### Statistical analysis

The two-sided Student’s t-test was used to test for differences, except for comparisons to negative controls, when the one-sided Student’s t-test was used.

## Supplementary Material

Supplementary Figures

Supplementary Table

## References

[1] Birney E, Stamatoyannopoulos JA, Dutta A, Guigó R, Gingeras TR, Margulies EH, Weng Z, Snyder M, Dermitzakis ET, Thurman RE, Kuehn MS, Taylor CM, Neph S, et al, and Children’s Hospital Oakland Research Institute. Identification and analysis of functional elements in 1% of the human genome by the ENCODE pilot project. Nature. 2007; 447:799–816. 10.1038/nature05874 17571346 PMC2212820

[2] Cech TR, Steitz JA. The noncoding RNA revolution-trashing old rules to forge new ones. Cell. 2014; 157:77–94. 10.1016/j.cell.2014.03.008 24679528

[3] Peschansky VJ, Wahlestedt C. Non-coding RNAs as direct and indirect modulators of epigenetic regulation. Epigenetics. 2014; 9:3–12. 10.4161/epi.27473 24739571 PMC3928183

[4] Brown CJ, Ballabio A, Rupert JL, Lafreniere RG, Grompe M, Tonlorenzi R, Willard HF. A gene from the region of the human X inactivation centre is expressed exclusively from the inactive X chromosome. Nature. 1991; 349:38–44. 10.1038/349038a0 1985261

[5] Hung T, Chang HY. Long noncoding RNA in genome regulation: prospects and mechanisms. RNA Biol. 2010; 7:582–85. 10.4161/rna.7.5.13216 20930520 PMC3073254

[6] Lamond AI, Spector DL. Nuclear speckles: a model for nuclear organelles. Nat Rev Mol Cell Biol. 2003; 4:605–12. 10.1038/nrm1172 12923522

[7] Derrien T, Johnson R, Bussotti G, Tanzer A, Djebali S, Tilgner H, Guernec G, Martin D, Merkel A, Knowles DG, Lagarde J, Veeravalli L, Ruan X, et al. The GENCODE v7 catalog of human long noncoding RNAs: analysis of their gene structure, evolution, and expression. Genome Res. 2012; 22:1775–89. 10.1101/gr.132159.111 22955988 PMC3431493

[8] Iyer MK, Niknafs YS, Malik R, Singhal U, Sahu A, Hosono Y, Barrette TR, Prensner JR, Evans JR, Zhao S, Poliakov A, Cao X, Dhanasekaran SM, et al. The landscape of long noncoding RNAs in the human transcriptome. Nat Genet. 2015; 47:199–208. 10.1038/ng.3192 25599403 PMC4417758

[9] http://www.lncrnadb.org.

[10] Glass D, Viñuela A, Davies MN, Ramasamy A, Parts L, Knowles D, Brown AA, Hedman AK, Small KS, Buil A, Grundberg E, Nica AC, Di Meglio P, et al, and MuTHER consortium. Gene expression changes with age in skin, adipose tissue, blood and brain. Genome Biol. 2013; 14:R75–14. 10.1186/gb-2013-14-7-r75 23889843 PMC4054017

[11] Zahn JM, Poosala S, Owen AB, Ingram DK, Lustig A, Carter A, Weeraratna AT, Taub DD, Gorospe M, Mazan-Mamczarz K, Lakatta EG, Boheler KR, Xu X, et al. AGEMAP: a gene expression database for aging in mice. PLoS Genet. 2007; 3:e201. 10.1371/journal.pgen.0030201 18081424 PMC2098796

[12] Kumar S, Millis AJ, Baglioni C. Expression of interleukin 1-inducible genes and production of interleukin 1 by aging human fibroblasts. Proc Natl Acad Sci USA. 1992; 89:4683–87. 10.1073/pnas.89.10.4683 1584804 PMC49147

[13] Coppé JP, Patil CK, Rodier F, Sun Y, Muñoz DP, Goldstein J, Nelson PS, Desprez PY, Campisi J. Senescence-associated secretory phenotypes reveal cell-nonautonomous functions of oncogenic RAS and the p53 tumor suppressor. PLoS Biol. 2008; 6:2853–68. 10.1371/journal.pbio.0060301 19053174 PMC2592359

[14] Mori Y, Hatamochi A, Arakawa M, Ueki H. Reduced expression of mRNA for transforming growth factor beta (TGF beta) and TGF beta receptors I and II and decreased TGF beta binding to the receptors in in vitro-aged fibroblasts. Arch Dermatol Res. 1998; 290:158–62. 10.1007/s004030050282 9558492

[15] Fazio MJ, Olsen DR, Kuivaniemi H, Chu ML, Davidson JM, Rosenbloom J, Uitto J. Isolation and characterization of human elastin cDNAs, and age-associated variation in elastin gene expression in cultured skin fibroblasts. Lab Invest. 1988; 58:270–77. 2831431

[16] http://www.ncbi-nlm.nih.gov/refseq.

[17] Rinn JL, Kertesz M, Wang JK, Squazzo SL, Xu X, Brugmann SA, Goodnough LH, Helms JA, Farnham PJ, Segal E, Chang HY. Functional demarcation of active and silent chromatin domains in human HOX loci by noncoding RNAs. Cell. 2007; 129:1311–23. 10.1016/j.cell.2007.05.022 17604720 PMC2084369

[18] Perez RL, Ritzenthaler JD, Roman J. Transcriptional regulation of the interleukin-1beta promoter via fibrinogen engagement of the CD18 integrin receptor. Am J Respir Cell Mol Biol. 1999; 20:1059–66. 10.1165/ajrcmb.20.5.3281 10226077

[19] Basak C, Pathak SK, Bhattacharyya A, Mandal D, Pathak S, Kundu M. NF-kappaB- and C/EBPbeta-driven interleukin-1beta gene expression and PAK1-mediated caspase-1 activation play essential roles in interleukin-1beta release from Helicobacter pylori lipopolysaccharide-stimulated macrophages. J Biol Chem. 2005; 280:4279–88. 10.1074/jbc.M412820200 15561713

[20] Shen F, Hu Z, Goswami J, Gaffen SL. Identification of common transcriptional regulatory elements in interleukin-17 target genes. J Biol Chem. 2006; 281:24138–48. 10.1074/jbc.M604597200 16798734

[21] Miyazawa K, Mori A, Yamamoto K, Okudaira H. Transcriptional roles of CCAAT/enhancer binding protein-beta, nuclear factor-kappaB, and C-promoter binding factor 1 in interleukin (IL)-1beta-induced IL-6 synthesis by human rheumatoid fibroblast-like synoviocytes. J Biol Chem. 1998; 273:7620–27. 10.1074/jbc.273.13.7620 9516466

[22] Casola A, Garofalo RP, Jamaluddin M, Vlahopoulos S, Brasier AR. Requirement of a novel upstream response element in respiratory syncytial virus-induced IL-8 gene expression. J Immunol. 2000; 164:5944–51. 10.4049/jimmunol.164.11.5944 10820277

[23] Tian B, Nowak DE, Jamaluddin M, Wang S, Brasier AR. Identification of direct genomic targets downstream of the nuclear factor-kappaB transcription factor mediating tumor necrosis factor signaling. J Biol Chem. 2005; 280:17435–48. 10.1074/jbc.M500437200 15722553

[24] Kolomeichuk SN, Bene A, Upreti M, Dennis RA, Lyle CS, Rajasekaran M, Chambers TC. Induction of apoptosis by vinblastine via c-Jun autoamplification and p53-independent down-regulation of p21WAF1/CIP1. Mol Pharmacol. 2008; 73:128–36. 10.1124/mol.108.039750 18094076

[25] Pan D, Pan LZ, Hill R, Marcato P, Shmulevitz M, Vassilev LT, Lee PW. Stabilisation of p53 enhances reovirus-induced apoptosis and virus spread through p53-dependent NF-κB activation. Br J Cancer. 2011; 105:1012–22. 10.1038/bjc.2011.325 21863032 PMC3185941

[26] Kwong KY, Literat A, Zhu NL, Huang HH, Li C, Jones CA, Minoo P. Expression of transforming growth factor beta (TGF-beta1) in human epithelial alveolar cells: a pro-inflammatory mediator independent pathway. Life Sci. 2004; 74:2941–57. 10.1016/j.lfs.2003.08.048 15051419

[27] Kähäri VM, Chen YQ, Bashir MM, Rosenbloom J, Uitto J. Tumor necrosis factor-alpha down-regulates human elastin gene expression. Evidence for the role of AP-1 in the suppression of promoter activity. J Biol Chem. 1992; 267:26134–41. 1281483

[28] Kuang PP, Berk JL, Rishikof DC, Foster JA, Humphries DE, Ricupero DA, Goldstein RH. NF-kappaB induced by IL-1beta inhibits elastin transcription and myofibroblast phenotype. Am J Physiol Cell Physiol. 2002; 283:C58–65. 10.1152/ajpcell.00314.2001 12055073

[29] Bose B, Soriani M, Tyrrell RM. Activation of expression of the c-fos oncogene by UVA irradiation in cultured human skin fibroblasts. Photochem Photobiol. 1999; 69:489–93. 10.1111/j.1751-1097.1999.tb03317.x 10212582

[30] Vile GF, Tanew-Ilitschew A, Tyrrell RM. Activation of NF-kappa B in human skin fibroblasts by the oxidative stress generated by UVA radiation. Photochem Photobiol. 1995; 62:463–68. 10.1111/j.1751-1097.1995.tb02369.x 8570706

[31] Asschert JG, Vellenga E, Ruiters MH, de Vries EG. Regulation of spontaneous and TNF/IFN-induced IL-6 expression in two human ovarian-carcinoma cell lines. Int J Cancer. 1999; 82:244–49. 10.1002/(SICI)1097-0215(19990719)82:2<244::AID-IJC15>3.0.CO;2-N 10389759

[32] Jiang W, Liu Y, Liu R, Zhang K, Zhang Y. The lncRNA DEANR1 facilitates human endoderm differentiation by activating FOXA2 expression. Cell Reports. 2015; 11:137–48. 10.1016/j.celrep.2015.03.008 25843708 PMC7721200

[33] Wang X, Arai S, Song X, Reichart D, Du K, Pascual G, Tempst P, Rosenfeld MG, Glass CK, Kurokawa R. Induced ncRNAs allosterically modify RNA-binding proteins in cis to inhibit transcription. Nature. 2008; 454:126–30. 10.1038/nature06992 18509338 PMC2823488

[34] Abdelmohsen K, Panda A, Kang MJ, Xu J, Selimyan R, Yoon JH, Martindale JL, De S, Wood WH 3rd, Becker KG, Gorospe M. Senescence-associated lncRNAs: senescence-associated long noncoding RNAs. Aging Cell. 2013; 12:890–900. 10.1111/acel.12115 23758631 PMC3773026

[35] Gloss BS, Dinger ME. The specificity of long noncoding RNA expression. Biochim Biophys Acta. 2016; 1859:16–22. 10.1016/j.bbagrm.2015.08.005 26297315

[36] Fagerberg L, Hallström BM, Oksvold P, Kampf C, Djureinovic D, Odeberg J, Habuka M, Tahmasebpoor S, Danielsson A, Edlund K, Asplund A, Sjöstedt E, Lundberg E, et al. Analysis of the human tissue-specific expression by genome-wide integration of transcriptomics and antibody-based proteomics. Mol Cell Proteomics. 2014; 13:397–406. 10.1074/mcp.M113.035600 24309898 PMC3916642

[37] Strausberg RL, Feingold EA, Grouse LH, Derge JG, Klausner RD, Collins FS, Wagner L, Shenmen CM, Schuler GD, Altschul SF, Zeeberg B, Buetow KH, Schaefer CF, et al, and Mammalian Gene Collection Program Team. Generation and initial analysis of more than 15,000 full-length human and mouse cDNA sequences. Proc Natl Acad Sci USA. 2002; 99:16899–903. 10.1073/pnas.242603899 12477932 PMC139241

[38] Wang P, Xue Y, Han Y, Lin L, Wu C, Xu S, Jiang Z, Xu J, Liu Q, Cao X. The STAT3-binding long noncoding RNA lnc-DC controls human dendritic cell differentiation. Science. 2014; 344:310–13. 10.1126/science.1251456 24744378

[39] Ng SY, Bogu GK, Soh BS, Stanton LW. The long noncoding RNA RMST interacts with SOX2 to regulate neurogenesis. Mol Cell. 2013; 51:349–59. 10.1016/j.molcel.2013.07.017 23932716

[40] Liu B, Sun L, Liu Q, Gong C, Yao Y, Lv X, Lin L, Yao H, Su F, Li D, Zeng M, Song E. A cytoplasmic NF-κB interacting long noncoding RNA blocks IκB phosphorylation and suppresses breast cancer metastasis. Cancer Cell. 2015; 27:370–81. 10.1016/j.ccell.2015.02.004 25759022

[41] Lopez-Pajares V, Qu K, Zhang J, Webster DE, Barajas BC, Siprashvili Z, Zarnegar BJ, Boxer LD, Rios EJ, Tao S, Kretz M, Khavari PA. A LncRNA-MAF:MAFB transcription factor network regulates epidermal differentiation. Dev Cell. 2015; 32:693–706. 10.1016/j.devcel.2015.01.028 25805135 PMC4456036

[42] McCarty G, Loeb DM. Hypoxia-sensitive epigenetic regulation of an antisense-oriented lncRNA controls WT1 expression in myeloid leukemia cells. PLoS One. 2015; 10:e0119837. 10.1371/journal.pone.0119837 25794157 PMC4368825

[43] Herriges MJ, Swarr DT, Morley MP, Rathi KS, Peng T, Stewart KM, Morrisey EE. Long noncoding RNAs are spatially correlated with transcription factors and regulate lung development. Genes Dev. 2014; 28:1363–79. 10.1101/gad.238782.114 24939938 PMC4066405

[44] Franklin CC, McCulloch AV, Kraft AS. In vitro association between the Jun protein family and the general transcription factors, TBP and TFIIB. Biochem J. 1995; 305:967–74. 10.1042/bj3050967 7848298 PMC1136352

[45] Guermah M, Malik S, Roeder RG. Involvement of TFIID and USA components in transcriptional activation of the human immunodeficiency virus promoter by NF-kappaB and Sp1. Mol Cell Biol. 1998; 18:3234–44. 10.1128/MCB.18.6.3234 9584164 PMC108905

[46] Yap KL, Li S, Muñoz-Cabello AM, Raguz S, Zeng L, Mujtaba S, Gil J, Walsh MJ, Zhou MM. Molecular interplay of the noncoding RNA ANRIL and methylated histone H3 lysine 27 by polycomb CBX7 in transcriptional silencing of INK4a. Mol Cell. 2010; 38:662–74. 10.1016/j.molcel.2010.03.021 20541999 PMC2886305

[47] Stanulis-Praeger BM, Gilchrest BA. Effect of donor age and prior sun exposure on growth inhibition of cultured human dermal fibroblasts by all trans-retinoic acid. J Cell Physiol. 1989; 139:116–24. 10.1002/jcp.1041390117 2708450

