# Supplementary material for: The long non-coding RNA FLJ46906 binds to the transcription factors NF-κB and AP-1 and regulates expression of aging-associated genes
Source: Aging (Albany NY). 2018 Aug 17;10(8):2037–50. doi: 10.18632/aging.101528 (PMC6128423; doi:10.18632/aging.101528)
Supplement: Supplementary Figures [file aging-10-101528-s001.pdf]

SUPPLEMENTARY FIGURES

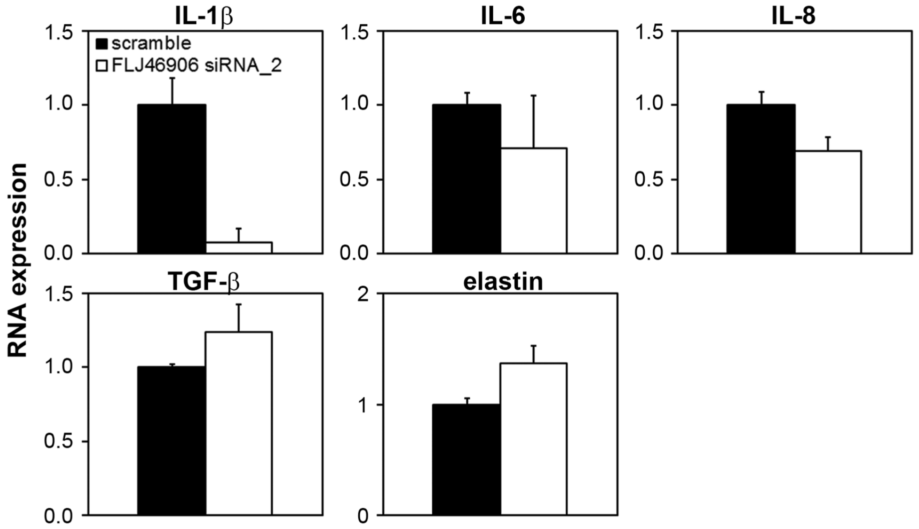

Supplementary Figure 1. Aging-associated genes are regulated by the lncRNA FLJ46906. Baseline expression of *IL1B* (interleukin-1β), *IL6* (interleukin-6), *CXCL8* (interleukin-8), *TGFB* (transforming growth factor-b), and *ELN* (elastin), as determined by qPCR, is altered by knockdown of FLJ46906 using a siRNA (siRNA 2) that is different from the one used for the experiments presented in figure 3A (n = 3, mean ± SD, p = 0.002 for IL-1β, 0.014 for IL-8, 0.017 for elastin, and n.s. for IL-6 and TGF-β).

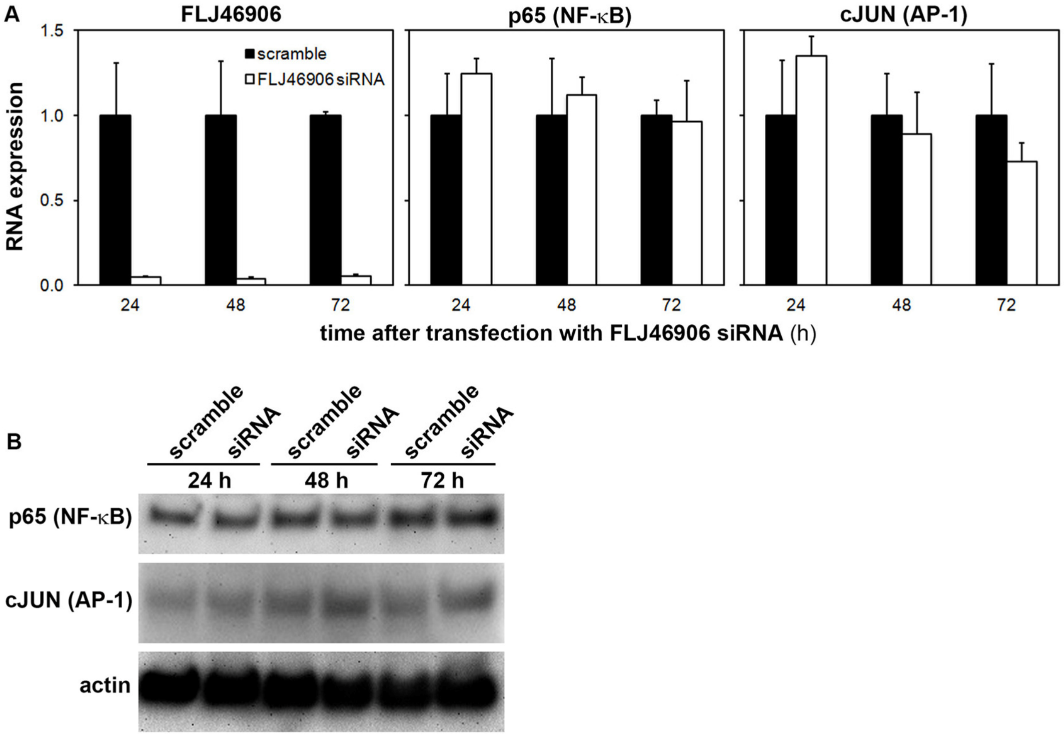

Supplementary Figure 2. The lncRNA FLJ46906 does not affect the expression of NF-κB and AP-1. (**A**) Efficient knockdown of FLJ46906 does not affect the mRNA expression of the NF-κB component p65 or of the AP-1 component cJUN (n = 3, mean ± SD, p = n.s.). (**B**) Knockdown of FLJ46906 does not affect the protein expression of the NF-κB component p65 or of the AP-1 component cJUN (Western blotting, actin = loading control).

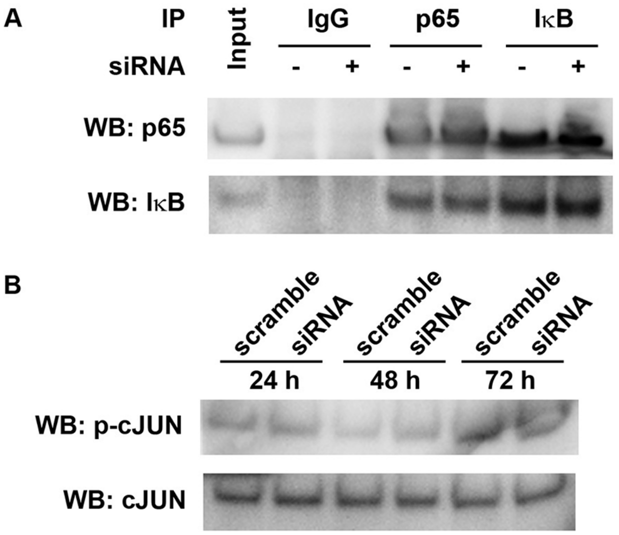

Supplementary Figure 3. The lncRNA FLJ46906 does not affect release of NF-κB from IκB or the phosphorylation of AP-1. (**A**) Immunoprecipitation (IP): 72 hours after knockdown of FLJ46906 (siRNA +) the amount of recovered IκB with pull-down of p65 (NF-κB) or the amount of p65 after pull-down of IκB are not changed, as compared to samples transfected with scrambled siRNA (siRNA -). WB = Western blot. This experiment was repeated once with similar results. (**B**) Western blot (WB): The levels of phosphorylated cJUN (AP-1) or of total cJUN do not change 24, 48, or 72 hours after knockdown of FLJ46906 (siRNA), as compared to samples transfected with scrambled siRNA (scramble). This experiment was repeated once with similar results.

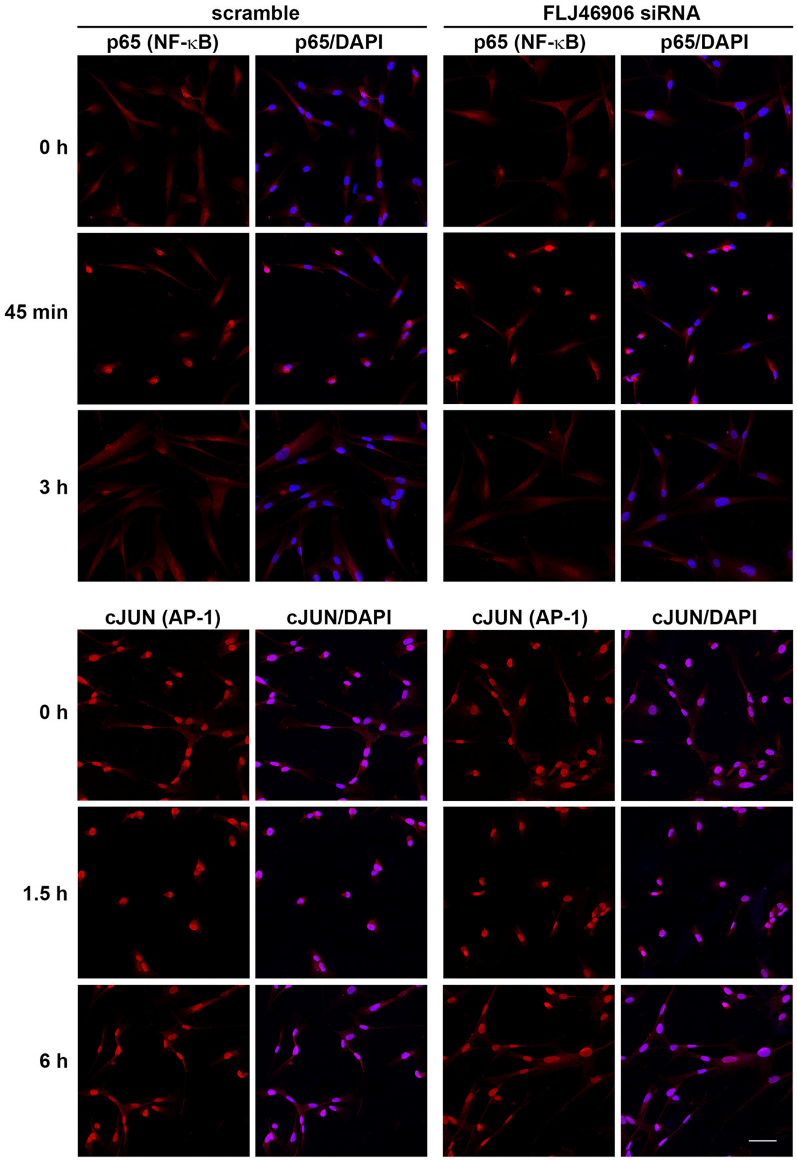

Supplementary Figure 4. The lncRNA FLJ46906 does not affect translocation of NF-κB or of AP-1 following irradiation with longwave ultraviolet light (UVA). Following irradiation with UVA (200 kJ/m^2), immunostaining showed translocation of p65 (NF-κB) from the cytosol to the nucleus within 45 min and back to the cytosol within 3 hours. Knockdown of FLJ46906 did not change these traffic patterns. Similar trafficking was observed with cJUN (AP-1), which were also not affected by knockdown of FLJ46906. Scale bar = 5 µm.

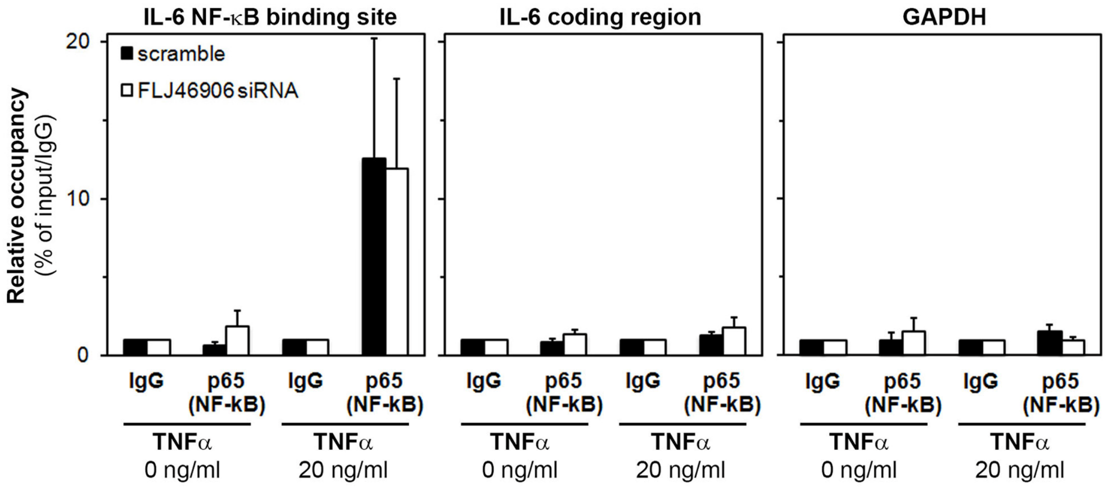

Supplementary Figure 5. The lncRNA FLJ46906 does not affect recruitment of NF-κB to its binding site on the IL-6 promoter. A ChIP assay using an antibody against p65 (NF-κB) was performed to measure binding of p65 to the DNA of the NF-κB binding site on the IL-6 promoter (left panel), to the DNA of the IL-6 coding region (negative control; middle panel), and to DNA of the GAPDH coding region (negative control; right panel). Binding of p65 was strongly induced 30 minutes after incubation with TNF-α (20 ng/ml). Knockdown of FLJ46906 did not reduce the binding of p65 to its binding site on the IL-6 promoter (left panel). n = 3 independent experiments with duplicate samples each, mean ± SEM.
