# Supplementary material for: The long non-coding RNA FLJ46906 binds to the transcription factors NF-κB and AP-1 and regulates expression of aging-associated genes
Source: Aging (Albany NY). 2018 Aug 17;10(8):2037–50. doi: 10.18632/aging.101528 (PMC6128423; doi:10.18632/aging.101528)
Supplement: Supplementary Table [file aging-10-101528-s002.pdf]

Supplementary Table 1. Primers used.

Gene	Direction	Sequence	Company
Expression assay			
CCDC28A	Forward	AACAGCCAGTGACTCCAATCTG	Thermo Fisher Scientific
	Reverse	TGGAACATCTTGTGCATCTGCC	Thermo Fisher Scientific
JUN	Forward	ACTCGGACCTCCTCACCTCG	Thermo Fisher Scientific
	Reverse	GATGTGCCCGTTGCTGGACT	Thermo Fisher Scientific
COL1A1	Forward	GTGAGAGAGGTCGCCCTGGA	Thermo Fisher Scientific
	Reverse	CCCGGCAGCACCAGTAGC	Thermo Fisher Scientific
CXCL8	Forward	GAGTGGACCACACTGCGCC	Thermo Fisher Scientific
	Reverse	ACCCTCTGCACCCAGTTTTCC	Thermo Fisher Scientific
ECT2L	Forward	ACCTCTGGACTAACAAGCAACGTC	Thermo Fisher Scientific
	Reverse	CCACTTTGGCCACTTGCATCC	Thermo Fisher Scientific
ELN	Forward	GGCTGCCAAGTACGGAGTGG	Thermo Fisher Scientific
	Reverse	AACCCAAACTGGGCGGCTTT	Thermo Fisher Scientific
FLJ46906	Forward	GTTGAGTGCCTGTGTGTTGGG	Thermo Fisher Scientific
	Reverse	TCGGGGAGGAACAAAGCTCC	Thermo Fisher Scientific
FLJ46906 siRNA 2	Forward	sequence not available, proprietary information	Qiagen
	Reverse	sequence not available, proprietary information	Qiagen
IL1B	Forward	TCGAGGCACAAGGCACAACA	Thermo Fisher Scientific
	Reverse	TCACTGGCGAGCTCAGGTACT	Thermo Fisher Scientific
IL6	Forward	AAGCCAGAGCTGTGCAGATGAG	Thermo Fisher Scientific
	Reverse	CGTCAGCAGGCTGGCATTTG	Thermo Fisher Scientific
LOC100507462	Forward	AAGCTGACGCAGCAAAAGGC	Thermo Fisher Scientific
	Reverse	GCGGAAAAGCCTCAGTGTCA	Thermo Fisher Scientific
MMP1	Forward	TGTCTCACAGCTTCCCAGCG	Thermo Fisher Scientific
	Reverse	CCGCTTTTCAACTTGCCTCCC	Thermo Fisher Scientific
MMP14	Forward	sequence not available, proprietary information	Qiagen
	Reverse	sequence not available, proprietary information	Qiagen
MMP3	Forward	sequence not available, proprietary information	Qiagen
	Reverse	sequence not available, proprietary information	Qiagen
MMP9	Forward	sequence not available, proprietary information	Qiagen
	Reverse	sequence not available, proprietary information	Qiagen
NHSL1	Forward	CAAGTGCCCGGTGGTTCAGT	Thermo Fisher Scientific
	Reverse	CACAGGGGTGAGTGCTGTGG	Thermo Fisher Scientific
RELA	Forward	GCAGGCTATCAGTCAGCGCA	Thermo Fisher Scientific
	Reverse	TCAGGTCGTAGTCCCCACGC	Thermo Fisher Scientific
RPL27	Forward	ATCGCCAAGAGATCAAAGATAA	Thermo Fisher Scientific
	Reverse	TCTGAAGACATCCTTATTGACG	Thermo Fisher Scientific

TGFB	Forward	sequence not available, proprietary information	Qiagen
	Reverse	sequence not available, proprietary information	Qiagen
RNA binding assay			
FLJ46906	Forward	GTTGAGTGCCTGTGTGTTGGG	Thermo Fisher Scientific
	Reverse	TCGGGGAGGAACAAAGCTCC	Thermo Fisher Scientific
U1	Forward	ATACTTACCTGGCAGGGGAG	Thermo Fisher Scientific
	Reverse	CAGGGGGAAAGCGCGAACGCA	Thermo Fisher Scientific
ChIP assay			
IL6 NF-kB binding site	Forward	CACCCTCACCCTCCAACAAA	Thermo Fisher Scientific
	Reverse	TTCTCTTTCGTTCCCGGTGG	Thermo Fisher Scientific
IL6 coding region	Forward	TGGACAGGTATGGCCAGAGA	Thermo Fisher Scientific
	Reverse	CAGAGAGGGAAAAGGCCCTG	Thermo Fisher Scientific
GAPDH	Forward	CCATGTTGCAACCGGGAAGG	Thermo Fisher Scientific
	Reverse	AGGAGCGCAGGGTTAGTCAC	Thermo Fisher Scientific
